# An assessment of cultural values contributing to antiretrovial therapy adherence among patients on antiretroviral therapy adherence among patients

**DOI:** 10.1186/1742-4690-9-S1-P83

**Published:** 2012-05-25

**Authors:** Kaona AD Frederick, Sikaona Lenganji, Miti Esnart, Siziya Seter

**Affiliations:** 1 Mwengu Social and Health Research Centre, Ndola, France

## Background

The study was conducted in 2007, in the Northern Province of Zambia, to assess the impact of cultural values contributing to ARV non-compliance among elderly patients afflicted by AIDS. Providing care for sick child, loss of income by the family, caring for the orphans, seeking treatment were studied. Parental experiences and impact of caregiving during the time of child illness that led to poor compliance are examined.

## Methods

A community-based survey was conducted in three randomly selected catchment areas of Nakonde, where 250 out of 682 patients receiving ARVs within the twelve months period, were recruited through the District's Health Management Board Zonal Health Centre. All patients were interviewed using a pre-tested structured questionnaire, consisting of: Socio-demographic characteristics, Socio-economic factors, Knowledge about HIV transmission and prevention. Cultural beliefs regarding ARVs in Zambia and the social taboos surrounding treatment and actual drug adherence were observed.

## Results

Most male AIDS patient respondents tended to be older and more educated than the female patient respondents. Overall, 32.6% of the patients stopped taking their medication. There were 49.1% of the females and 23% of the males, who reported that AIDS patients stopped taking their medication within the first 4 months of commencing treatment. Age, marital status and educational levels were not significantly associated with compliance. The major factors leading to non-compliance included patients beginning to feel better (55% and 48.6%), lack of knowledge on the benefits of completing a course (25.7%), running out of drugs at home (25.4%) and ARVs drugs too strong (20.1% and 20.2%). There was a significant difference [OR = 1.66, 95% CI 1.23, 2.26] in HIV knowledge, with more males than females reporting shaking hands as a means for HIV transmission, after adjusting for age, marital status and educational levels.

## Conclusion

This study established that 32% of AIDS patients failed to comply with AIDS drug taking regimen once they started feeling better. Providing means and knowledge for primary caregiver to administer effective drugs and ARVs or to prevent and treat opportunistic infections, TB and pneumonia, would help reduce caregivers distress.

